# A Community-Led Central Kitchen Model for School Feeding Programs in the Philippines: Learnings for Multisectoral Action for Health

**DOI:** 10.9745/GHSP-D-21-00391

**Published:** 2022-12-21

**Authors:** Vanessa T. Siy Van, Carmina P. Siguin, Andrew C. Lacsina, Lean Franzl Yao, Zarah G. Sales, Normahitta P. Gordoncillo, Leslie Advincula-Lopez, Joselito T. Sescon, Eden Delight P. Miro

**Affiliations:** a Health Sciences Program, Ateneo de Manila University, Quezon City, Philippines.; b Community Welfare, Wellness, and Well-being Laboratory, Ateneo de Manila University, Quezon City, Philippines.; c Department of Mathematics, Ateneo de Manila University, Quezon City, Philippines.; d Institute of Human Nutrition and Food, University of the Philippines Los Baños, Laguna, Philippines.; e Development Studies Program, Ateneo de Manila University, Quezon City, Philippines.; f Department of Economics, Ateneo de Manila University, Quezon City, Philippines.

## Abstract

In devolved governments like the Philippines, local government units (LGUs) must be engaged to develop and coordinate responses to tackle the multisectoral problem of childhood undernutrition. However, current Philippine nutrition interventions, such as decentralized school feeding programs (SFPs), generally rely on the national government, public school teachers, or the private sector for implementation, with mixed results. The central kitchen model for SFPs was developed by 2 Philippine nongovernmental organizations and facilitated large-scale in-school feeding through community multisectoral action. This case study documented coordination processes in February 2018 for 1 urban city and 1 rural province—the model’s earliest large-scale implementation sites—that contributed to its institutionalization and sustainability. Data from 24-hour dietary recalls with 308 rural and 310 urban public school students and household surveys with their caregivers showed undernutrition was an urgent problem. Enabling factors and innovative local solutions were explored in focus group discussions with 160 multisector participants and implementers in health care, education, and government, as well as volunteers, parents, and central kitchen staff. The locally led and operated central kitchens promoted community ownership by embedding volunteer pools in social networks and spurring demand for related social services from their LGU. With the LGU as the face of implementation, operations were sustained despite political leadership changes, fostering local government stewardship over nutrition. Leveraging national legislation and funding for SFPs and guided by the Department of Education’s standards for SFP eligibility, LGUs had room to adapt the model to local needs. Central kitchens afforded opportunities for scale-up and flexibility that were utilized during natural disasters and the coronavirus disease (COVID-19) pandemic. The case demonstrated empowering civil society can hold volunteers, local implementers, and local governments accountable for multisectoral action in decentralized settings. The model may serve as a template for how other social services can be scaled and implemented in devolved settings.

## Abstract (precis)

A central kitchen model for implementing a school feeding program in the Philippines increased demand for local health interventions, empowered civil society, and held local governments accountable for multisectoral action in a decentralized government.

## Teaser

**Key Findings:**

- Combining the logistics of a centralized in-school program with decentralized implementation to improve the diets and nutritional status of school-age children, a community-supported central kitchen model prepared and packed meals for schools.
- The model’s success was attributed to financial sustainability institutionalized at the national level through laws, operational sustainability maintained by local government units (LGUs), strong community support, mobilization from volunteers, and embedding of the program in the city’s and municipalities’ social networks.
- Community involvement in central kitchen operations translated into volunteer accountability and program ownership that organized local interests and enabled community members to demand related health, education, and social services from their local elected officials, contributing to their sustained interest in participating in the program.

**Key Implications:**

- In decentralized settings, national health agendas and services can successfully be devolved to LGUs when they are afforded decision-making space to adapt programs according to local needs and resources with national support.
- When developing health interventions, nongovernmental organizations should consider factors influencing program sustainability such as the involvement of multisectoral community-led action from inception and the country’s government structure.

## BACKGROUND

Childhood malnutrition is an immediate, lifetime, and intergenerational concern. Undernutrition increases risk of diseases,^1^ hampers cognitive development,^2^ and leads to considerable economic losses.^3^ Inadequate nutrition has also been linked to poor education outcomes,^4^ a crucial element of poverty reduction.^5^ Globally, over 618 million people experienced hunger in 2019.^6^ The prevalence of undernourishment has since increased from 8.0% before the coronavirus disease (COVID-19) pandemic to 9.8% in 2021.^6^ Chronic hunger leads to negative childhood nutritional outcomes, such as being underweight for one’s age, and stunting, being short for one’s age.^7^ Today more than 55% of people facing hunger live in Asia,^8^ and 24.7% of children in Southeast Asia are considered stunted, higher than the global average.^7^

The Philippines is a lower-middle-income country in Southeast Asia,^9^ where high poverty rates have led to slow improvements in childhood stunting and underweight prevalence over the last 30 years.^10^ Currently, 25.5% of school-aged children are underweight and 24.9% are stunted.^11^ The poor are especially vulnerable, as 83.2% of households in the poorest income quintile are food insecure, and the prevalence of both underweight and stunting are 5% higher in rural compared to urban areas.^11^

To address the problem of malnutrition, in 2018, the Philippines ratified Republic Act 11037, referred to as the National Feeding Program (NFP) Law, which institutionalized an NFP for undernourished children aged 3–5 years in public day care programs and a school-based feeding program (SFP) for children in kindergarten through grade 6 in public schools.^12^ The NFP Law aimed to improve children’s nutritional status, classroom attendance, enrollment rate, and classroom participation. By enacting this law, the government formally standardized and scaled up feeding initiatives that the Department of Education (DepEd) had implemented since 1997, and tasked the DepEd and the Department of Social Welfare and Development (DSWD) with implementing the SFP in public schools and supplemental feeding programs in day care centers, respectively.

The NFP provides at least 1 fortified meal to undernourished children in public schools and day care centers over a minimum period of 120 days. Meals include components with micronutrient supplementation such as fortified milk and iodized salt. Children are deemed undernourished if they fall below the World Health Organization (WHO) Child Growth Standards (CGS) for wasting (body-mass-index [BMI]-for-age below -2 Z-score line).^13^ Program beneficiaries are also given health examinations to measure their weight and height before and after participation. SFP beneficiaries are deemed rehabilitated if their BMI-for-age is between the -2 and +2 Z-score lines of the WHO CGS.^13^ In 2019, nearly one-fifth of all Philippine school-aged children participated in the government’s NFP.^11^

However, there remains a lack of consensus on the impact of these SFPs. While literature acknowledges enhancement in caloric and micronutrient intake, these changes do not always correspond to anthropometric improvements.^14^^–^^16^ Prior research also found positive impacts on student attendance and participation; however, evidence on cognition and academic achievement has been inconclusive.^14^^,^^15^ Inconsistencies have been attributed to a lack of evidence-based program design and the varying quality of SFPs globally.^17^ In low- and middle-income countries (LMICs), SFP evaluations also tend to be inadequately designed, usually less than 1 year without follow-up. Given this time frame, there were no substantial effects on conventional anthropometric indicators such as height-for-age, weight-for-age, and BMI-for-age Z-scores.^17^

The modality of implementation affects outcomes as well. Based on service delivery, SFPs are broadly classified into 2 groups: (1) in-school feeding, when children eat the meals in school; or (2) take-home rations, when families are given food for children attending school regularly.^18^ While in-school service delivery faces more logistical challenges, the positive impact of take-home rations is diminished by monitoring issues. Literature has shown that an intrahousehold “flypaper effect” exists among poorer families, in which the take-home meal may be shared with other family members and not just the child, negating the potential caloric gains from the social transfer.^19^^,^^20^

Another service delivery classification is based on the program’s back-end structure. In the traditional centralized model, a government or nonprofit organization distributes food to schools. In contrast, the decentralized model, or “home-grown school meals,” devolves the procurement and operations to schools that purchase food locally from farmers.^21^ However, these decentralized programs are complex, requiring coordination from multiple sectors and the cooperation of various stakeholders.^22^^–^^24^

For decades, decentralized, in-school feeding programs have been implemented by DepEd, private organizations, and nongovernmental organizations (NGOs) in the Philippines with mixed results. Previous evaluations found operational issues, such as the quality of meals and measuring equipment, human resource shortages, and household characteristics, contributed to students’ regression and program unsustainability and stressed the need for alternative interventions and innovations to traditional feeding models.^25^^–^^27^

The central kitchen model we describe in this study is an innovation of SFPs that blends the streamlined logistics of a centralized SFP with the community-building benefits of a decentralized SFP.^28^ The central kitchen model facilitates large-scale feeding by procuring, preparing, and packing meals for multiple schools in 1 kitchen staffed by community volunteers. Recent research suggests that community-participatory interventions improve primary health care and nutrition outcomes.^29^^–^^31^ The program in this study was used as a template by national legislators for the NFP Law, which has provisions for LGU and private-sector involvement (Sections 7 and 8), as well as the creation of a national nutrition information system overseen by the National Nutrition Council (Section 6).^12^ In the law’s implementing rules and regulations, signed in 2021, the centralized kitchen model is presented as the main modality of NFP implementation (Section 5.2.3).^32^

The NFP is a school-based feeding program that provides at least 1 fortified meal to undernourished children in public school and day care centers.

The central kitchen model blends the streamlined logistics of a centralized SFP with the community-building benefits of a decentralized SFP to facilitate large-scale feeding.

Local support for these programs is critical in the Philippines, where the government is decentralized and the national agenda for health and nutrition is operationalized by local government units (LGUs).^33^ Assessments of local health systems have found that after decentralization, LGU investment in population-level health care services declined,^34^ as elected local chief executives (LCEs) may not prioritize public health.^35^^,^^36^ However, LGUs’ roles in mediating policies from various national government agencies (NGAs)^37^ highlight their potential for multisectoral planning and coordination to address the multidimensional determinants of malnutrition.^38^ Though the literature has explored challenges to multisectoral action for health in LMICs, successful models of local-led multisectoral models are scarcer.^39^^–^^41^

This study presents 2 such LGUs, 1 urban and 1 rural, where the adoption of a locally developed central kitchen model for SFPs has withstood shocks and changes in political leadership and improved children’s health and communities' civic engagement outcomes. We examine the common challenges faced by implementers and key factors that contributed to the program’s success and sustainability, which could inform NFP Law implementation and may be applied to other community-based multisector inventions.

## CENTRAL KITCHEN MODEL DESCRIPTION

### Design and Innovation

In 2011, the Ateneo Center for Educational Development (ACED), an education NGO, developed a template for Blueplate for Better Learning, a large-scale feeding program for public elementary school students. The template was inspired by a partnership with Jollibee Foundation’s *Busog, Lusog, Talino* feeding program, where parent volunteers prepared meals for undernourished children. ACED also learned from Akshaya Patra Foundation’s Mid-day Meal Program for underserved public school students in India, which established central kitchens to cook meals and deliver them to several schools in the area.^42^

The central kitchen model innovated upon these programs in the following 3 ways.

1. To adapt it to the Philippine context, which has a devolved form of government,^33^ ACED took the existing concept of SFPs and repositioned it as a multisectoral project by creating a new way for NGAs (e.g., DepEd and DSWD), NGOs (e.g., ACED and later Gawad Kalinga [GK]), and LGUs to synergistically deliver national services, with the LGU acting as the focal point to reach intended beneficiaries.
2. Process innovations in preparing and packaging food enabled cost-effective and replicable scale-up of central kitchens to provide food for entire cities or municipalities. In central kitchens, standardized food preparation and packing took place to maximize resources and avoid waste. This innovation sought to alleviate the burden of traditional SFPs on teachers, who procured ingredients, cooked, fed, and cleaned up after students, all of which detracted focus from teaching. Though DepEd models envisioned parent volunteers fulfilling these roles, volunteerism was often inconsistent or unsustainable.
3. ACED’s central kitchen model focused on community empowerment through community ownership, with local volunteers integral to central kitchen and feeding activities.

Central kitchens enable standardized, cost-effective food preparation and packaging to maximize resources and avoid waste.

In 2014, Gawad Kalinga, a community development foundation, partnered with ACED to expand the use of the model across the country, culminating with central kitchens as the main modality of SFPs in the NFP Law (Figure).

**FIGURE:**
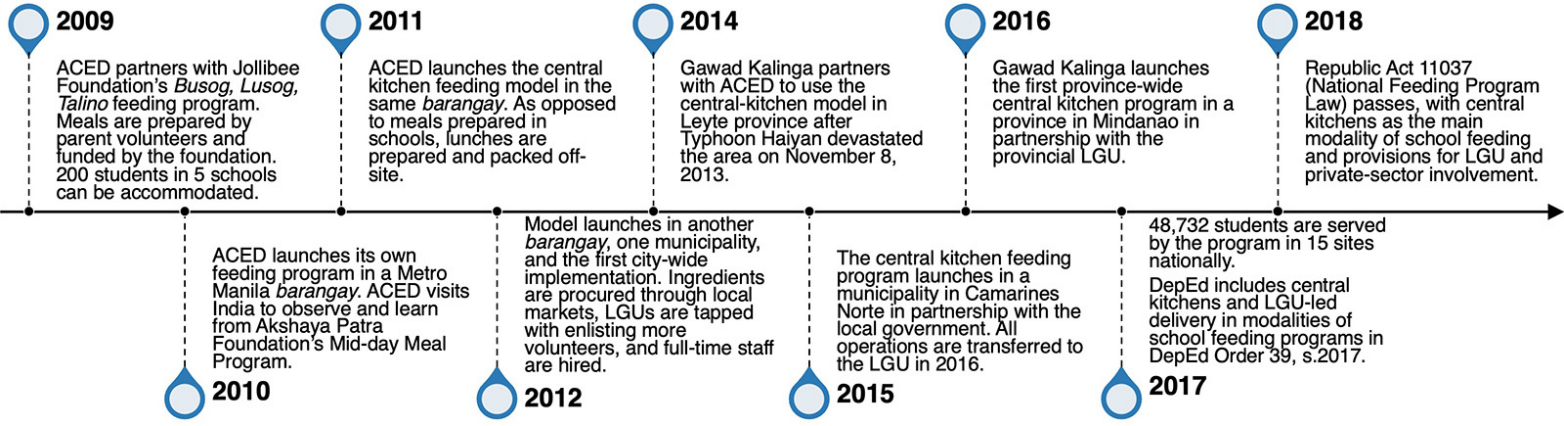
Timeline of Philippine Central Kitchen Model Evolution Abbreviations: ACED, Ateneo Center for Educational Development; DepEd, Department of Education; LGU, local government unit.

Initially, ACED and GK partnered with willing LGUs for 1-year pilots. The 2 NGOs provided training and technology, as well as building a back-end support infrastructure such as procurement, liquidation, and monitoring systems. They also helped orient teachers and kitchen staff for operations. After the pilot, LGUs’ were evaluated on their cohesion, leadership, and volunteerism. Though the model has more than 100 central kitchens around the country, many of these remain privately funded and operated. Pilots were deemed successful only in sites where LGUs demonstrated both interest and capability for implementation, where capability was the ability to mobilize the community and secure the buy-in of local stakeholders in a process called “social preparation,” as volunteers sustained kitchen and feeding operations.

After the pilot year (2011 in the urban site and 2015 in the rural site), the successful LGUs proposed the program to DepEd, which funded the program for subsequent years through their SFP budget (Table 1). The program undergoes regular evaluation and iterative improvements are implemented based on feedback and recommendations. Though the template is strictly followed, decision-making space is deliberately left for local implementers to adapt the model to variations in local contexts.

**TABLE 1.** Stakeholders and Roles in the Philippine Central Kitchen SFP Model

Stakeholder	Role	Description
National level		
Department of Education	Implementing agency of SFPs in public schools	Fund public schools to conduct SFPs Monitor activities for SFPs (receive budget and liquidation reports, participant growth) Enforce standards for program eligibility, minimum duration, and rehabilitation DepEd nurses identify undernourished students to be included in feeding programs
Department of Social Welfare and Development	Implementing agency of supplemental feeding programs in day care centers	Fund day care centers for feeding activities Monitor feeding program (receive budget and liquidation reports, participant growth reports)
Local level		
Local government unit	Implements the central kitchen model of SFP in their respective localities: provincial-level LGU in the rural site and city-level LGU in the urban site	Provide additional funds for central kitchens or school feeding operations from their internal revenue allotment (micronutrient food fortification, inclusion of milk and fruits in meals, and medical check-ups) Provide additional human and capital resources for logistical needs of the central kitchen (e.g., hire feeding coordinators to prepare reports on student attendance, grades, and nutrition for monitoring) Coordinate with local health sector to conduct medical examinations and flag beneficiaries whose nutrition status did not improve after participation Identify and contract local food suppliers for central kitchens Mobilize civil society groups and community members to volunteer in SFP
Public schools	Administer SFP to undernourished public school students	Provide venue for feeding operations Teachers oversee school feeding and coordinate feeding volunteers Communicate student feedback about the program to LGU and central kitchen Inform parents about the feeding program and children’s beneficiary status Provide vehicle to transport meals from central kitchen to schools
City or municipal health office	Monitors SFP participants’ nutritional status	Conduct medical examinations of students in public schools Conduct medical examinations at the end of the feeding cycle for nonrehabilitated beneficiaries
Nongovernmental organizations	Capacity building and innovation for the SFP	Design the central kitchen model Develop and innovate feeding cycle menu Develop web-based integrated nutrition database for different levels of stakeholders Train volunteer staff for central kitchen, orient teachers and LGUs about the central kitchen model Advocated for widespread adoption of the central kitchen model in the NFP Law
Central kitchen	Site for meal preparation and packing	Prepare inventory and financial reports for monitoring Instruct and oversee volunteers on food preparation and packing
Volunteers	Food preparation and feeding Consists of parents of both beneficiaries and nonbeneficiaries, members of parent-teacher associations, and local people’s organizations	One set of volunteers work in central kitchens to prepare ingredients, pack meals, and clean containers after feeding Another set of volunteers carry out feeding operations (serving feeding meals, ensuring participants finish their meals, cleaning up meal containers) Educate other members of the community about SFP and volunteering
Local food suppliers	Provide meal ingredients	Contracted by LGU Deliver fresh ingredients to central kitchens daily

Abbreviations: DepEd, Department of Education; LGU, local government unit; NFP, National Feeding Program; SFP, school feeding program.

The current roles of each stakeholder are presented in Table 1. As this case study focuses on program operations in elementary schools and not day care centers, we mention DSWD only briefly.

### Program Objectives

Multisectoral coordination among schools, parents, the community, LGUs, NGAs, and NGOs is crucial to meet the following program objectives.^43^

- To strengthen the program to scale up nationally, develop an effective and sustainable large-scale comprehensive feeding program that can reach all malnourished children in public elementary schools in the Philippines
- To provide access to quality foods, change dietary habits, and improve health and education outcomes:
  - To reduce rates of malnutrition, stunting, and wasting among participating children
  - To lay a foundation for lifelong healthy eating based on favorable experiences, the acquisition of sufficient skills, and confidence in one’s capacity to practice a healthy lifestyle
- To encourage maximum community support for the feeding program and empower the school community and other stakeholders to take the lead role in the program
- To encourage a whole-of-society approach through multisectoral investment to improve the health and well-being of students and their families

Multisectoral coordination among schools, parents, community, LGUs, and NGOs is crucial to develop an effective and sustainable large-scale comprehensive feeding program.

### Implementation

Concrete mechanisms to facilitate coordination were built into the program even before implementation. First, the NGOs consulted with local stakeholders to understand their capabilities and set expectations. Then, central kitchen staff were trained in food preparation, kitchen maintenance, and management, such as inventory, logistics, volunteer management, and preparing reports for monitoring and transparency. A project coordinator from the NGO guided the process during the pilot phase. Project coordinators monitored daily feeding operations and liaised with central kitchen staff, school administrators, and local government. Unlike in the traditional DepEd SFP, feeding coordinators monitored volunteers in schools and compiled feeding and nutrition reports, kitchen staff monitored volunteer attendance and inventory, and LGU staff handled procurement and liquidation reports. All reports were processed by the LGU and sent to DepEd for accountability.

During implementation, national leadership through DepEd and local implementation through LGUs allowed operations to run continuously throughout the school year. Leveraging existing policies for school feeding, DepEd set standards for program eligibility, targets for rehabilitation, and funds SFPs. Through their local budgets, LGUs hired feeding coordinators to bridge the oversight role initially filled by ACED and permanent staff within the LGU that were responsible for local nutrition programs’ budget and procurement. The provincial LGU in this study also established an annual Hunger Summit with NGOs, inviting all stakeholders, from DepEd to community members, to be transparent about program performance for the year.

At the national level, ACED and GK adopted the Hunger Summit to generate awareness of malnutrition among LCEs and legislators and share best practices from the central kitchen SFP model. This summit led to the incorporation of the LGU-led central kitchen model in DepEd’s SFP operations in 2017 and the NFP Law in 2018.

Annually, the NGOs continue to conduct a quality control assessment in all central kitchens. However, because their goal is to transfer monitoring and evaluation to LGUs and DepEd, they developed a web-based data collection and monitoring system and began training project coordinators on its use as a crucial input in LGUs’ and DepEd’s evaluation of SFPs, though this has not yet been completed.

### Student Eligibility

Student beneficiaries are identified through the same standards as DepEd’s SFPs. If their BMI-for-age falls below the −2 Z-score line based on the WHO CGS, they are considered wasted and enrolled in the program. At the end of the 130-day feeding cycle, 10 days longer than the standard DepEd SFP, a beneficiary is considered rehabilitated if their BMI-for-age is between the −2 and +2 Z-score lines of the WHO CGS. The program aims to rehabilitate 90% of beneficiaries. Although not originally prescribed by DepEd or the NGOs, a unique feature of the program is that through LGU coordination with the local health office, nonrehabilitated beneficiaries are given health check-ups to determine whether other health factors have contributed to their malnutrition.

A unique feature of the program is that through LGU coordination with the local health office, nonrehabilitated beneficiaries are given health check-ups to determine whether other health factors contribute to their malnutrition.

## METHODS

For this cross-sectional case study, we employed a mixed-methods approach to examine the Philippine central kitchen model for SFPs. We chose 1 city in Metro Manila and 1 province in Mindanao for the study setting. The province is comprised of 11 municipalities, which include an urban town or city and its surrounding rural areas. In 2012, the urban site of the study became the first city-wide implementation of the program through the city LGU, while the rural site of the study was the first to implement it province-wide in 2016 through the provincial LGU.

### Data Collection

To provide context for the program setting, elementary school students were randomly sampled from all 41 public schools of the urban site, and 60 of the 352 public schools of the province (Table 2), using a list of SFP beneficiaries provided by DepEd. As the schools were dispersed across each implementation site, schools were assumed independent from one another. Schools matched sampled beneficiaries with a random nonbeneficiary of the same grade level. However, the list of city beneficiaries provided was 2 years outdated, with most beneficiaries rehabilitated and taken off the SFP. Only students with the same SFP status at the time of the study were included, leading to a decreased number of urban study participants. From about 70,000 public school students, 310 were sampled from the urban site, and 308 of about 120,000 students from the rural site. We excluded day care facilities from the sampling frame for this study. However, in the rural site, children aged 3–5 years were enrolled in elementary schools, so we included this age group in the sample.

**TABLE 2:** Characteristics of Participants in the Philippine Central Kitchen SFP Study, by Site Type and Beneficiary Status

	Rural (n=308)		Urban (n=310)	
	SFP Beneficiary	SFP Nonbeneficiary	SFP Beneficiary	SFP Nonbeneficiary
Age, years				
3–5	35	28	0	1
6–9	63	73	19	115
10–12	46	48	26	119
13–18	12	3	11	19
Total	156	152	56	254

Abbreviation: SFP, school feeding program.

Data collection took place in February 2018 for the city and October 2019 for the province. Nutritionist-enumerators conducted 24-hour dietary recalls 3 times (2 weekdays and 1 weekend) within the week following standard practice to accurately compute their average daily dietary intake.^44^ A structured interview with the child’s caregiver and the child recorded all food and beverages consumed the previous day. Intakes were then converted into nutrient values. Household food security was captured in a concurrent survey with caregivers, as the study ensured that no students came from the same household. The Household Food Insecurity Access Scale,^45^ developed by the U.S. Agency for International Development, was used for this measurement.

To understand program operations and multisectoral coordination in its implementation, participants from different sectors were recruited for guided focus groups. Focus group discussions (FGDs) with parents, educators, city and provincial local government officials, government employees from local offices of national agencies, health care workers, and central kitchen staff (Table 3) provided insight into the community-level impact and perception of the program, as well as factors contributing to its successes or challenges. Information on coordination mechanisms among implementers was triangulated with the types of forms and reports shared among NGOs, schools, and government offices.

**TABLE 3:** Characteristics of Focus Group Discussion Participants in the Philippine Central Kitchen SFP Study

**Sector**	**Rural**	**Urban**	**Total **
Parents	5	18	23
Education sector	20	15	35
Health sector	5	10	15
Local government	14	21	35
Central kitchens	29	21	50
Nongovernmental organizations	1	1	2
Total	74	86	160

### Data Analysis

Descriptive statistics from food recalls and household surveys provided context for respondents’ nutritional adequacy and household food security status in both sites. Qualitative thematic analysis on the FGD transcripts aimed to identify trends, agreeing or conflicting ideas, and factors contributing to the successes or shortcomings of the program from multisectoral perspectives.^46^ In the Results section, we present translated quotes and redact portions of translations that may lead to identification of the participants. A review of secondary literature from similar programs and country contexts verified whether the derived themes contributed to program outcomes.

### Ethical Approval

Ethical approval was granted by the Ateneo University Research Ethics Office, and all participants gave their written informed consent. The sites were deidentified to ensure confidentiality of local government officials and school administrators participating in the FGDs, who could be identified by their location.

## RESULTS

### Program Implementation Setting

Undernutrition was a pressing issue at both implementation sites. Among the households surveyed, only 9.77% of rural households and 39.03% of urban households were food secure. Based on the Philippine Dietary Reference Intakes,^47^ the caloric intake of children in surveyed households was inadequate for their age and sex. Rural children reached only a median of 58% daily caloric adequacy and urban children reached 88% daily caloric adequacy, with 100% signifying adequate caloric consumption.

According to a report by ACED for the urban site,^48^ between 86% and 91% of beneficiaries were rehabilitated and drop-out rates remained below 4% of beneficiaries from 2012 to 2018 (Table 4). This contrasts with a 2016 evaluation of DepEd’s traditional SFPs, which found about 62% of beneficiaries had been rehabilitated, below DepEd’s target of 70% of beneficiaries attaining normal weight.^25^ Parents, educators, health care workers, and local government officials also observed decreases in the number of beneficiaries after each program cycle, advocating for the continual expansion of the program.

**TABLE 4.** Year-End Nutrition Status of Central Kitchen Feeding Beneficiaries in the Philippines^48^

**School Year**	**Beneficiaries**	**Rehabilitated, No. (%)**	**Not Rehabilitated, No. (%)**	**Dropped Out, No. (%) **
2012–2013	5,265	4,556 (86.53)	614 (11.66)	95 (1.81)
2013–2014	8,542	7,553 (88.42)	899 (10.52)	90 (1.06)
2014–2015	7,116	6,442 (90.53)	539 (7.57)	135 (1.90)
2015–2016	6,409	5,742 (89.6)	589 (9.19)	78 (1.21)
2016–2017	5,756	4,971 (86.37)	560 (9.73)	225 (3.90)
2017–2018	5,726	5,027 (87.80)	557 (9.73)	142 (2.47)

The feeding process was similar to those of traditional SFPs.^49^ According to an educator in a focus group, feeding promoted other contiguous habits, such as incorporating vegetables in diets, handwashing, and cleaning up after oneself. Feeding coordinators noted aversion to vegetables at the beginning of the feeding cycle, though beneficiaries adjusted after a few weeks.

Though these individual-level effects cannot directly be attributed to the central kitchen model, the program has standardized nutritionally balanced meals developed by nutritionists to ensure children they were exposed to a variety of vegetables and age-appropriate meal portions. Under the traditional decentralized SFPs, teachers explained that they were not able to consistently follow DepEd’s recommended recipes due to a lack of available ingredients, fluctuations in market prices, and difficulties in meal preparation on top of their teaching responsibilities.

Based on the program’s secondary goal to improve education outcomes, in the FGDs, a local government official and an educator reported that program participation increased school attendance and participation because students no longer needed to find food before classes or leave campus during lunch hours, especially for rural beneficiaries whose homes were far from school. These findings were similar to previous evaluations of Philippine SFPs, which showed that attendance improved after participation.^25^^,^^49^ However, neither parents nor educators could determine whether there was a clear impact on students’ grades and participation in extracurricular activities, and attendance was not systematically investigated in this study.

More pressing was the challenge of regression during the summer months when schools were not in operation. The following sections discuss how the respective communities overcame issues of nutritional regression, volunteer commitment, and operational sustainability.

Three interconnected mechanisms facilitated the program’s successful implementation in the 2 sites: community involvement and program ownership, local government stewardship and coordination, and program scale-up and sustainability.

### Community Involvement and Program Ownership

Implementation of the SFP necessitated the creation of volunteer networks and grassroots support that increased demand for more community-based health interventions and protected program sustainability from threats of political interference. Community participation and roles varied in the 2 LGUs, as LGUs in the Philippines are given some discretionary funds and administrative capacity under the country’s Local Government Code of 1991 (Republic Act 7160).^33^ The decision-making space of LGUs was recognized by ACED and GK, allowing for variation in program implementation across sites.

Rural communities were less dense and geographically dispersed across the large province. Consequently, 10 of the 11 municipalities in the rural site had 1 central kitchen established in contrast to the single central kitchen of the city. The exception was 1 municipality, which required 3 central kitchens because of its size. While kitchen staff were drawn from the community, they were considered government employees and paid a salary, relying less on informal community ties to ensure responsibilities were fulfilled. Nonetheless, community buy-in to the importance of nutrition, which began with the SFP, facilitated local engagement in the program: feeding volunteers were uncompensated but consistently assisted in the schools. Though the program was a provincial-level, then municipal-level initiative, barangay (the smallest unit of local government) officials and residents volunteered to deliver meals to their respective schools. Volunteer local government employees also supported a provincial program, which provided aid to nonrehabilitated beneficiaries’ families over the summer break, to mitigate household-level causes of malnutrition.

In the urban site, 98% of volunteers were uncompensated, and 66% of volunteers did not have children participating in the SFP. However, the central kitchen could reliably assign 50 volunteers from their pool of about 600 to arrive for the day’s operations. The sustainability of the city’s volunteer pool over 6 years was attributed to ingraining volunteer operations within community relationships. In the initial phases of implementation, SFP volunteers were drawn from local people’s organizations, in particular, women’s groups supporting the LCE. As the program matured, recruitment from civil society groups waned. In 2018, 29% of volunteers joined through people’s organizations, while 51% were recruited through information from friendships and neighborhood relationships. Urban volunteers were recognized for their participation through a year-end party organized by the LGU.

*Why do you volunteer? You don’t have children in the feeding [program]*. —Interviewer

*Because in the beginning, the officers at [civil-society association] are my clique, but even if I wasn’t an officer, [they said] “Oh, sis, come on!” So there. Helping out. Until they left already. When someone else was in charge and needed volunteers, you know, experienced ones. “Okay, yeah,” was what I said…so I already got used to helping out.* — Parent, urban FGD

The sustainability of the city’s volunteer pool over 8 years was attributed to ingraining volunteer operations within community relationships.

Communities’ active participation in local policy encouraged demand for more integrated health services. LGUs responded by subsidizing medical check-ups and treatment for nonrehabilitated beneficiaries, as DepEd found that students whose nutritional status did not improve after the feeding cycle usually had underlying medical concerns, according to health workers in the focus group. LGUs also made structural investments in social services through programs sustained by community volunteerism. SFP kitchens were used in disaster relief operations, as the country is vulnerable to tropical storms. The urban LGU continued feeding operations during summer classes, as the SFP was integrated into an ambitious multifaceted educational investment program including educational reform, parenting seminars, and building physical and technological infrastructure. To generate another source of ingredients for central kitchens, the provincial LGU launched home and school gardening programs for household income generation and food production, and locally sourced ingredients for feeding.

People’s roles in the success of their own programs created a sense of ownership that withstood changes in political leadership. Since its pilot in 2011, the city SFP has persisted through 4 terms and 2 mayors, while the provincial SFP has operated since 2016. This culminated in local ordinances that codified local priority and support for the SFP, according to local government officials in focus groups, ensuring these programs’ implementation regardless of who is elected to local office.

### Local Government Stewardship and Coordination

The program underwent an iterative process of planning, implementation, and evaluation with multisectoral input, which helped overcome challenges faced by traditional feeding models. Public school feeding was originally under the purview of DepEd, and operations were decentralized to individual schools; however, schools faced difficulties coordinating with the centralized NGA. School staff identified beneficiaries, bought and cooked ingredients, and submitted progress reports and liquidation forms for reimbursement. In both urban and rural settings, this additional uncompensated work was assigned to teachers, who reported being unable to focus on teaching and paying out-of-pocket when ingredients were insufficient or unavailable in the market. DepEd’s hierarchical bureaucratic processes delayed reimbursements and incentivized schools to decline feeding programs.

*Before the [DepEd SFP], I fed [students] through the help of the parents. So, every 3 times a week, we cooked. We got our funds from the [school] canteen…Later, the [DepEd SFP] came. We were given a budget of 15-pesos or 16-pesos per child. So, last year—2 years ago—I think, I was asked if I would accept the SBFP again. I really didn’t want anymore because it was so tiring being the [SBFP] coordinator. Because I was the one who went to the market and I was the one who liquidated, and then if the parents I assigned didn’t come in or come here, I was the one who had to cook. So, it was really tiring, really tiring.* —Educator, rural FGD

When the centralized-kitchen model was first proposed, schools in the city perceived the LGU did not trust them to implement the program on their own, as one local government official reported. The LCE held dialogues with all public school administrators to onboard them. Following the successful pilot, the LGU then coordinated with DepEd to develop a unified work-and-financial plan, as each had unique liquidation and procurement protocols that necessitated a new system to interface between them. Through the LGUs’ efforts, initial ambivalence toward the program was overcome.

As another local government official stated, LGUs deliberately engaged other sectors to minimize threats to implementation. Though successful pilots were funded by DepEd in subsequent years of operation, LGUs augmented funds through their internal revenue allotment and contributed to building and renovating kitchens and hiring full-time kitchen staff. By mobilizing their civil society groups, LGUs created an initial pool of volunteers. They also responded to context-specific needs. To alleviate the burden of SFP implementation on teachers, the city LGU hired full-time feeding coordinators to conduct feeding and monitoring activities. The provincial LGU coordinated transportation at the barangay-level so teachers did not need to pick up or return meal containers. LGUs also played an important role in expectation setting and both formal and informal accountability. When some schools tasked feeding coordinators with teaching errands, they were reprimanded by the mayor and the practice was stopped. Volunteers, though unpaid, attend an annual seminar retreat organized by the LGU to refresh their training and raise morale. As such, stakeholders felt recognized and seen and were incentivized to perform better because they were being rewarded or sanctioned for their actions.

*You see your name has a blank everywhere. Then you feel ashamed moving forward [laughter]. I see my name is blank, I get ashamed to be absent.* —Central kitchen volunteer, urban FGD

*It’s unacceptable that you don’t find a solution. You need to find a solution because every day there are those [students] who need to eat.* —Central kitchen staff, rural FGD

LGUs deliberately engaged other sectors to minimize threats to implementation such as burden on teachers to feed and monitor and challenges in transporting meals.

Because the LGU sat at the intersection of different government agencies, the central kitchens were maximized across sectors. Coordinating with the DSWD’s city office, the central kitchens also provided universal feeding for the city’s 70 day care centers as an early childhood health intervention. Collaborating with the local health care sector, LGUs began to include micronutrient supplementation in the SFP. When class suspensions occurred, LGUs transported packed meals to shelters, detention centers, and health care workers, to prevent food waste. The LGUs remained receptive to feedback from parents, teachers, and implementation partners. FGD participants often mentioned the modification of the feeding menu for each locale, accounting for local market availability and familiarity with the ingredients used. For instance, tomato-based sauces had small amounts of added sugar or used Filipino-style sweet sauce popular in the country. In the rural site, higher energy requirements necessitated increasing rice portions. Given the limited availability of vegetables, local vegetables were used, and potatoes were substituted with sweet potatoes. Instead of mushroom sauce, the province used coconut milk with turmeric.

Students and parents initially complained about every meal being soup-based with finely chopped viands. Though research-based and nutritious, when packed with rice, the meals would resemble slop or porridge. Drier viands were quickly substituted, increasing the acceptability of the program.

*They [students] compare the food with what was there before. “At least the one before, our viands were delicious.” You could really see the viand, the beef and the contents…Because now, we chop [everything], ground beef.* —Educator, rural FGD

*Before it was like that, there were really those [meals] that the children didn’t want to eat, especially the food that was sweet, the one like the food of the elderly.* —Parent, urban FGD

### Scale-Up and Sustainability

Visible successes in multisectoral collaboration have fostered a culture of data sharing and evidence-based decision making among stakeholders. Respondents from all sectors proudly shared that their SFP protocols were based on research, making them trustworthy. Program data were not only collected from schools but also compared to program goals. Results and recommendations were presented to school principals, feeding coordinators, and kitchen managers semiannually by the LGU and NGOs.

*It’s not really an issue for us because of support. All the programs and projects of the LGU, we always support…Because we know that those are beneficial to us, to the children, to the community, to the parents. Just like another project they have that’s also ongoing, the [parent-teacher seminars]. We support the LGU because we know the results of it will benefit us too…Why not support, right?* —Educator, urban FGD

*Actually, Mayor looked forward to the [evaluation] study. Because we wanted [to know] how can we improve the feeding more. Because the problem of malnutrition does not originate from just a simple problem, right?* —Local government employee, urban FGD

Multisectoral involvement in the SFP had spurred investments in physical and social infrastructure that allowed the program to continue operations despite crises. Despite the shift to remote learning during the COVID-19 pandemic, central kitchen operations continued. In the provincial site, meals were delivered to barangays and distributed directly to households by barangay health workers, barangay nutrition scholars, and volunteers from the Sanggunian ng Kabataan (barangay youth councils).

In the city, the central kitchen provided meals to health care workers at the height of community lockdowns from March to May 2020, when mobility was limited to reduce the spread of COVID-19. Due to limitations on gathering sizes and community quarantines, central kitchen operations were reduced. Through the central kitchen channels, beneficiary children were initially given food packs and Nutribun, fortified bread distributed in DepEd’s usual SFP. However, full kitchen operations are planned to resume in November 2022 as all students in the country return to on-site classes.^50^

The central kitchen model eased the scaling-up process by facilitating LGU initiatives to implement universal feeding in economically disadvantaged areas, such as a relocated community in the city, and geographically isolated and disadvantaged areas in the province. This was enabled in part by economies of scale.^28^ DepEd sets each meal for SFPs at 16 Philippine pesos (PHP) (about US$0.31 in 2018 prices), for a total of PHP1,920 (US$37) per beneficiary for the 120-day feeding cycle.^25^ Through the central kitchen, the actual cost per meal was about PHP11.50 (US$0.23) per day, and PHP1,380 (US$27) per beneficiary for 120 days.

The central kitchen model eased the scaling up process by facilitating LGU initiatives to implement universal feeding in economically disadvantaged areas and geographically isolated and disadvantaged areas in the province.

The success of the central kitchen SFP model has prompted other localities to inquire about the program template for implementation in their own LGUs.

*Our visitors—they also benchmark against us—love shortcuts. Because immediately they want the root. “Where’s the root?” …They’re looking for the root of the problem. Why is it like that? Because in their [operations], there are volunteers, but it still failed*. — Central kitchen staff, urban FGD

In addition to the assistance from volunteers, a key contributor to success was the input received from multiple stakeholders, which led to improvements in existing feeding procedures as well as innovations applicable to other local government programs. For instance, implementers expressed a major concern about the need for a single database for feeding coordinators, principals, DepEd, ACED, and GK, with each having different levels of access to the data while maintaining children’s data privacy. This prompted ACED’s development of a web-based integrated nutrition database where schools input individuals’ nutrition data, DepEd’s local offices consolidate them, LGUs generate reports, and ACED provides recommendations for LGU interventions beyond SFP. User training for the application was conducted in 2019; the full pilot scheduled for 2020 was interrupted by the COVID-19 pandemic.

Table 5 summarizes the implementation results in both sites. Differences in the implementation experiences of the rural and urban sites are highlighted, as the model allowed for local modifications to adapt the program.

**TABLE 5:** Key Results From Pilot Implementation of the Philippine Central Kitchen SFP Model

	**Urban Site (City)**	**Rural Site (Province)**
Setting	Highly urbanized city in Metro Manila within the National Capital Region	Province in Mindanao comprising 11 municipalities, defined as an urban town or city and its surrounding rural areas
	First site city-wide implementation in 2012 city LGU	First site province-wide implementation in 2016 provincial LGU
Financing	Daily feeding operations funded by the DepEd under the budget for SFPs—later the budget for the National Feeding Program—reimbursing LGUs implementation costs (as opposed to reimbursing public schools as in the traditional DepEd model), upon submission of liquidation reports	
	Additional funds from LGUs’ internal revenue allotment to construct central kitchens and hire staff as necessary based on local conditions and needs	
Governance	LGUs established dialogue with school administrators to counter institutional distrust of feeding programs based on experience with traditional decentralized in-school feeding	
	LGUs coordinated with local health offices for student check-ups to determine eligibility	
	LGU coordinated with DepEd to create a streamlined working and financial plan used in succeeding implementation sites	LGU organized the Hunger Summit with NGOs, eventually adopted by DepEd to advocate for a national feeding program law
	LGUs codified the central kitchen model of SFP in local ordinances	
	Information summits and lobbying organized by NGOs and participating LGUs led to the national government incorporating the central kitchen model as the main modality of SFP in the Philippines in the National Feeding Program Law (Republic Act 11037)	
	LGUs initiated program modifications according to city or municipality context (see below)	
Physical infrastructure	1 central kitchen for the entire city	1 central kitchen for each of the 10 municipalities; the 11^th^ municipality built 3 central kitchens because of its size
Human resources	NGOs facilitate technology transfer: engage local chief executives, train kitchen staff on food preparation, and initially oversee operations during pilot through a project coordinator	
	Hired full-time feeding coordinators to accomplish daily reports on feeding and volunteer attendance	Teachers were feeding coordinators but focused on attendance and feeding reports as opposed to feeding students and handling liquidation
	Volunteers for feeding and kitchens were unpaid, but efforts were recognized through LGU-sponsored events	Feeding volunteers were uncompensated while kitchen staff were employed by the provincial LGU and paid a salary
	Volunteer recruitment initially from people’s organizations in support of the local chief executive	
	Volunteers mobilized through community relationships such as friendships, family, and neighbors; includes parents of program participants and nonparticipants, and nonparents	
Service delivery	Ingredients and recipes for the feeding menu were adapted to locally available vegetables, dietary (energy) requirements, and cultural tastes	
		Provincial and municipal LGUs engaged with barangay LGUs to help with meal deliveries since schools were more dispersed in the area
	Additional services using the central kitchens:	
	Extending feeding operations to summer school for beneficiary students	Local government employees can support food-insecure students during the summer
	LGUs subsidize health check-ups for nonrehabilitated SFP participants	
	Central kitchens used in disaster-relief operations for natural hazards such as typhoons	
	LGUs used central kitchens to implement universal feeding in disadvantaged areas	
	During the COVID-19 pandemic, food packs and fortified bread were distributed to households with beneficiaries	During the COVID-19 pandemic, meals were prepared in central kitchens and delivered directly to households with beneficiaries
Monitoring	Local data collected from schools and kitchens were consolidated and utilized by LGUs for quality improvement, then submitted to DepEd for their annual report to the Office of the President and Congress	

Abbreviations: COVID-19, coronavirus disease; DepEd, Department of Education; LGU, local government unit; NGO, nongovernmental organization; SFP, school feeding program.

## DISCUSSION

A community-supported central kitchen model for SFPs is a relatively novel form of technology transfer by local NGOs. The model combined in-school feeding with decentralized implementation in the Philippines, where LGUs have political and financial decision-making space.^33^ However, unlike traditional models, which devolve procurement and operations to individual schools, meals were prepared and packed by a central kitchen catering to all schools in a city or municipality. Our case study examined a multisectoral pilot intervention to improve the diets and nutritional status of low-income children at the local level through an analysis of the program’s facilitating factors, particularly how multisectoral coordination was achieved.

At the local level, implementation created and strengthened multisectoral collaborative networks between the communities, schools, and LGUs, and fostered a culture of evidence-based policy making and civil engagement. The model’s success was attributed to strong community support for the central kitchen, mobilized into a steady pool of volunteers, embedding the program in the city’s and municipalities’ social networks. Public participation in local policy making compelled local government investment in adjacent holistic health and nutrition interventions by rallying nonhealth sectors to create new systems for multisectoral collaboration. Operations were sustained despite changes in political leadership through both formal and informal accountability mechanisms facilitated by transparent monitoring and evaluation.

From the local to national level, vertical accountability was fostered by designating focal persons for feeding operations instead of increasing teachers’ workloads. Feeding coordinators collected feedback from schools, students, and volunteers, relaying information to the kitchens and LGUs. Daily operational reports were filled out by kitchen staff. Forms were collected by the LGU and submitted to DepEd. These were used in DepEd’s annual monitoring reports, submitted to and evaluated by the Office of the President and Congress as stipulated in the implementing rules and regulations of the NFP Law (Section 10).^32^

The central kitchen SFP model envisioned multisectoral, community-led action from its inception. While a wide body of research^40^^,^^41^^,^^51^^,^^52^ indicates governance for multisectoral action in LMICs is notoriously difficult, this case study presented 3 learnings to account for in the design and evaluation of multisectoral policies.

First, community-based interventions have been increasingly recognized as a vital component of health promotion. Community buy-in was crucial to sustaining interest in the program, as parent volunteers are necessary even in traditional SFPs. Community members, including parents of non-participants, were more inclined to volunteer in support of the program when it was attached to the LGU. As the program matured, social relationships sustained volunteer interest after a new LCE was elected. Civil participation brings nuanced knowledge, social trust, and formal and informal regulation to health programs.^53^^,^^54^ Community input is particularly relevant in LMICs, where interventions are rarely locally conceived and led, and policies may be incompatible with local structural contexts.^55^ Where community volunteers are able to organize and lead health initiatives, support and sustainability tend to be higher compared to programs led and implemented by external stakeholders.^56^ A key contributing factor is the creation of an enabling environment through community engagement, education, and mobilization,^57^ facilitating smoother implementation. Evidence from a volunteer community malnutrition intervention in Tajikistan points to volunteers’ ability to directly improve their own health outcomes while changing social behavior, indirectly improving health.^58^ By integrating volunteer networks into the social fabric of the city and municipalities where the SFP is implemented, an adequate number of staff prevented possible challenges to continuity,^59^ as volunteers felt their actions directly accountable to their respective communities.^60^

Community buy-in was crucial to sustaining interest in the program, as parent volunteers are necessary even in traditional SFPs.

Second, civil clamor for health interventions such as the SFP is important, especially in decentralized health systems like that of the Philippines where LCEs are elected officials who may not necessarily prioritize health.^61^ Compared to other decentralized LMICs, Philippine LCEs were found to have wide decision-making space,^62^ translating into discretionary power to prioritize agendas and allocate resources. The NGOs’ clear delegation of management to the LGU for the success of the pilot served as a political incentive for elected leaders to galvanize parents, community networks, and schools, and negotiate multisectoral arrangements with the local offices of other government agencies in the education, health, and social welfare sectors. These arrangements were instrumental in overcoming siloed performance and the inclination towards sector-specific achievements,^63^ borne from each sector’s own understanding^51^ of malnutrition and the feasible policy solutions it can unilaterally implement. These differing sectoral goals may be a potential source of conflict,^41^ as was illustrated by actors in the education sector initially hesitant to cede control of what they deemed were their responsibilities. However, LGUs’ use of well-defined operational plans that articulated sectors’ mutual gain from the SFP secured their commitment to providing support infrastructure beyond the central kitchen, leading both sites to overcome the problem of regression over the summer. This case study shows how health services can be ratified in the national agenda, funded through NGAs, and implemented by LGUs. Although LGUs could implement SFPs independently, partnering with DepEd and other NGAs augments financial resources and opportunities for synergy with other health policies, such as micronutrient fortification and health examinations.^64^

Third, strong leadership demonstrated by local leaders was complemented by their openness to feedback and support for evidence-based innovations to the model. While funding sustainability for the program is enshrined in national law,^12^ presenting LGUs as implementers aided in maintaining operational sustainability. As the program became associated with LCEs’ political performance, communities’ apparent involvement in the model necessitated attention to local health needs. In contrast, traditional authoritarian leadership styles were negatively associated with cohesion in cross-specialization teams.^59^ Though the lack of formal horizontal accountability mechanisms poses a challenge in multisectoral interventions,^65^ LGUs were able to overcome it with monitoring and information systems that enforced each implementer’s responsibility to the community and innovations for consolidated databases to swiftly identify implementation gaps.^66^^,^^67^ Moreover, NGOs and LCEs’ low tolerance for corruption and noncompliance reinforced necessary social sanctions that led to the program’s sustainability,^41^ despite natural hazards, disasters, and other shocks.

Strong leadership demonstrated by local leaders was complemented with an openness to feedback and support for evidence-based innovations to the model.

The roles played by communities in program advocacy, operations, and accountability emphasize the need for community-based interventions to promote local ownership and allow room for nuanced variations to bring about a sense of agency and empowerment. As the model helped inform major provisions in the NFP Law, the case study presents supporting evidence for the use of central kitchens in SFPs, continued engagement of LGUs in executing national policies, and the institutionalization of hiring and training focal persons to handle multisectoral, multilevel health programs when implementing the law in subsequent sites.

### Limitations

Given the study’s focus on community-led health interventions, only 2 implementation sites of the many central kitchens established by the NGOs were chosen. These were the biggest and first city- and province-wide operations, completely sustained by their local governments and communities. Though their experiences may not easily be generalizable to sites that did not manage to secure full community or LGU support, findings provided a picture of the confluence of factors necessary for success, as well as how challenges were overcome at the pilot, development, and mature phases of implementation. The inclusion of 1 urban and 1 rural site represented possible structural differences that may have affected implementation.

Survey data, particularly dietary recalls, were sensitive to recall bias.^68^ To improve accuracy, respondents were asked about only the previous day’s meals, and each household was visited thrice, nonconsecutively, within the week of data collection. The caloric equivalents of the 3 days were then averaged to increase the precision of the recalls according to standard techniques.^44^ Because urban SFP beneficiaries were limited by the outdated DepEd-provided list, only those who remained beneficiaries over the 2 years of lag were included. However, these data were used only to contextualize the study setting and validate how local implementers perceived the urgency of undernutrition. Future studies that focus on the quantitative effects of the model can compare its individual-level impacts with those of traditional feeding models.

As the research aimed to analyze the processes of a novel intervention involving multisectoral coordination, a full impact analysis comparing central kitchen model feeding meals with those of traditional SFPs was not undertaken. Qualitative data were more appropriate to understand coordination channels, accountability mechanisms, and program evolution in the different sites. As implementers such as local government officials and school administrators were included in FGDs, data may have been susceptible to social desirability bias.^69^ However, responses from 1 sector were verified against those of other sectors and official reports and documents. Though the researchers were unable to interview every implementer of the program, data collection from FGDs had reached a point of saturation and common themes could be identified from participants’ responses.^70^ Moreover, literature reviews were employed when necessary to provide more context for each sector’s tasks, policies, and capabilities.

## CONCLUSION

Locally led and operated central kitchens are a multisectoral investment that can spur health, education, and social welfare interventions. The experiences of 2 successful large-scale implementation sites present a model for improving diet and health, empowering civil society, and holding groups with a variety of interests accountable for multisectoral action in decentralized governments. Local-initiated innovations to traditional social programs increase acceptability and appropriateness. Operational sustainability was attributed to affording communities agency to provide feedback and gain ownership over program activities, embed volunteer pools in social networks, and organize demand for related services from their local politicians. Support and regulation from the national government in funding and accountability standards served as a foundation that enabled local governments to modify and augment the program according to their communities’ needs. Beyond the cost-effectiveness of constructing a large-scale central kitchen, the model institutionalized multisectoral coordination channels that may serve as a template for how other social services can be scaled and implemented in devolved settings.

## Notes

**Peer Reviewed**

**First published online:** December 5, 2022.
